# Plant-based diets, insulin sensitivity and inflammation in elderly men with chronic kidney disease

**DOI:** 10.1007/s40620-020-00765-6

**Published:** 2020-06-08

**Authors:** Ailema González-Ortiz, Hong Xu, Carla M. Avesani, Bengt Lindholm, Tommy Cederholm, Ulf Risérus, Johan Ärnlöv, Angeles Espinosa-Cuevas, Juan Jesús Carrero

**Affiliations:** 1 Department of Medical Epidemiology and Biostatistics (MEB), Karolinska Institutet, Nobels väg 12A, Box 281, 171 77 Stockholm, Sweden; 2 Nephrology and Mineral Metabolism Department, Instituto Nacional de Ciencias Médicas y Nutrición Salvador Zubirán, Mexico City, Mexico; 3 Division of Clinical Geriatrics, Department of Neurobiology, Care Sciences and Society, Karolinska Institutet, Stockholm, Sweden; 4 Renal Medicine and Baxter Novum, Clinical Science, Intervention and Technology, Karolinska Institutet, Stockholm, Sweden; 5 Clinical Nutrition and Metabolism, Dept. of Public Health and Caring Sciences, Uppsala University, Uppsala, Sweden; 6 Theme Ageing, Karolinska University Hospital, Stockholm, Sweden; 7 School of Health and Social Studies, Dalarna University, Falun, Sweden; 8 Division of Family Medicine and Primary Care, Department of Neurobiology, Care Sciences and Society (NVS), Karolinska Institutet, Alfred Nobels Allé 23, 14183 Huddinge, Sweden

**Keywords:** Fruit, Vegetable, Malnutrition, Protein-energy wasting, Potasssium, Restriction

## Abstract

**Background:**

In persons with CKD, adherence to plant-based diets is associated with lower risk of CKD progression and death, but underlying mechanisms are poorly characterized. We here explore associations between adherence to plant-based diets and measures of insulin sensitivity and inflammation in men with CKD stages 3–5.

**Methods:**

Cross-sectional study including 418 men free from diabetes, aged 70–71 years and with cystatin-C estimated glomerular filtration rate (eGFR) <60 mL/min/1.73m^2^ and not receiving kidney-specific dietetic advice. Information from 7-day food records was used to evaluate the adherence to a plant-based diet index (PBDi), which scores positively the intake of plant-foods and negatively animal-foods. Insulin sensitivity and glucose disposal rate were assessed with the gold-standard hyperinsulinemic euglycemic glucose clamp technique. Inflammation was evaluated by serum concentrations of C-reactive protein (CRP) and interleukin (IL)-6. Associations were explored through linear regression and restricted cubic splines.

**Results:**

The majority of men had CKD stage 3a. Hypertension and cardiovascular disease were the most common comorbidities. The median PBDi was 38 (range 14–55). Across higher quintiles of PBDi (i.e. higher adherence), participants were less often smokers, consumed less alcohol, had lower BMI and higher eGFR (P for trend <0.05 for all). Across higher PBDi quintiles, patients exhibited higher insulin sensitivity and lower inflammation (P for trend <0.05). After adjustment for eGFR, lifestyle factors, BMI, comorbidities and energy intake, a higher PBDi score remained associated with higher glucose disposal rate and insulin sensitivity as well as with lower levels of IL-6 and CRP.

**Conclusion:**

In elderly men with non-dialysis CKD stages 3–5, adherence to a plant-based diet was associated with higher insulin sensitivity and lower inflammation, supporting a possible role of plant-based diets in the prevention of metabolic complications of CKD.

**Graphic abstract:**

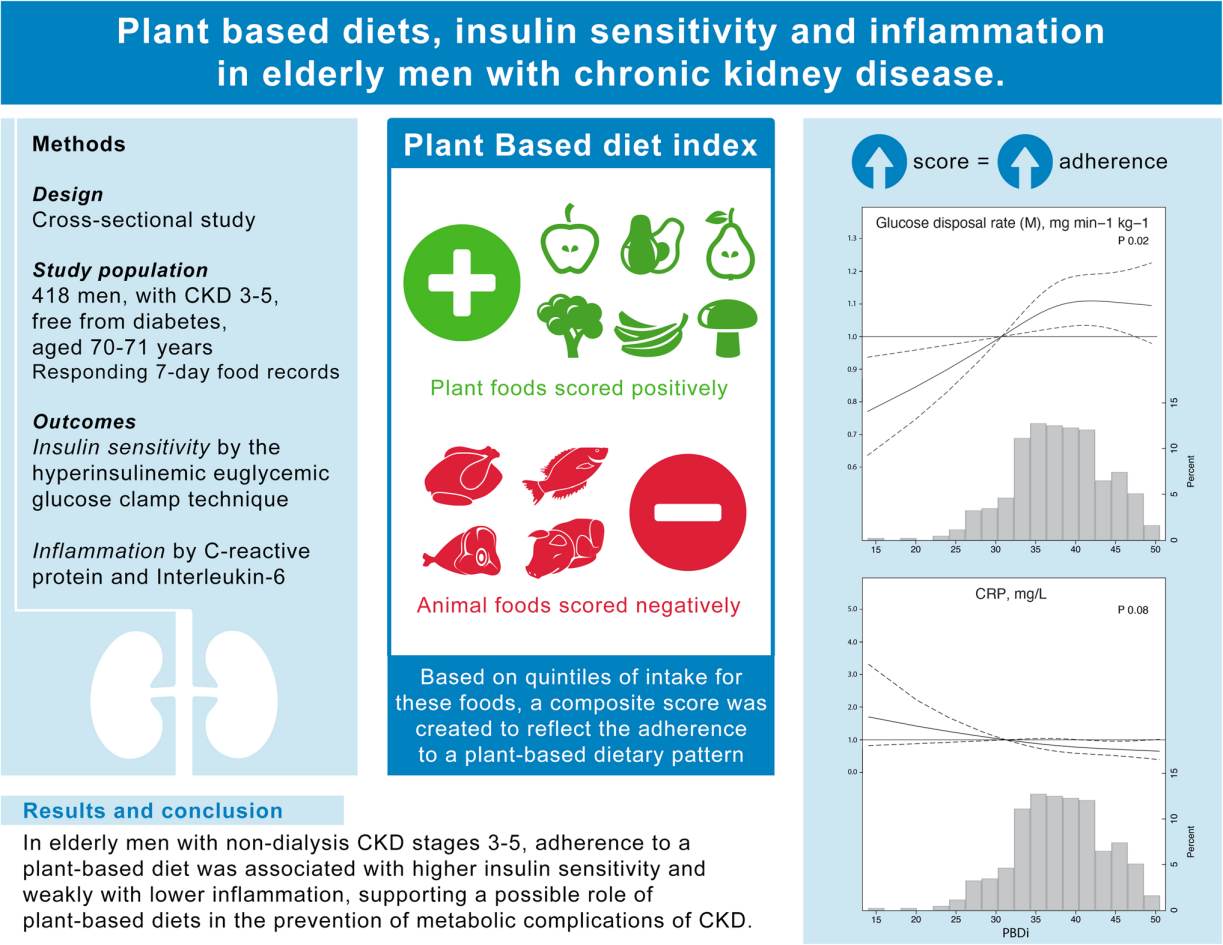

## Introduction

Dietary recommendations to treat chronic kidney disease (CKD) are focused on restricting dietary protein intake to avoid kidney overload and limiting the amount of nutrients that can accumulate into toxic levels (sodium, potassium, phosphorus) while ensuring sufficient energy intake [1, 2]. Without appropriate dietary counselling, such dietary restrictions can result in a diet of low food variety, often with a low intake of fruits and vegetables as a direct consequence of dietary potassium restriction [3, 4]. A number of recent observational studies suggest, however, that a more liberal consumption of plant-based foods in persons with CKD is associated with a lower risk of death and progression to end-stage kidney disease (ESKD) [5–7]. Mechanisms underlying these associations have, however, been poorly investigated.

One established mechanism by which increased intake of plant-foods may benefit CKD patients involves an overall reduction of dietary acid load [8], which in randomized trials lead to a better control of metabolic acidosis, reduction in urine excretion of angiotensinogen and estimated glomerular filtration rate (eGFR) preservation compared to usual care [9]. Plant-based diets have often been used as models for low-protein diets, which can delay the progression to ESKD and the need to start dialysis [10]. On the other hand, patients with CKD have progressively increased insulin resistance [11], and it has been proposed that a higher intake of plant foods may improve insulin sensitivity potentially through increasing the amount of short-chain fatty acids in plasma as a result of a higher fermentation of fiber by the microbiota [12, 13]. While prospective studies in persons with diabetes suggest that dietary patterns rich in animal foods and poor in plants associate with insulin resistance [14], the studies in patients with CKD are scarce.. If any, a small (n = 39 patients) 4-week trial [15] of fermentable fiber supplementation in persons with CKD G4 failed to observe changes in insulin resistance as estimated by the homeostatic model assessment (HOMA-IR). A third mechanisms by which plant-based diets may benefit persons with CKD is through the induction of lower systemic inflammation attributed to increased intake of fiber, phytochemicals, antioxidants and vitamins [16, 17]. Animal [18] and human studies in persons with CKD [19] show significant amelioration of oxidative stress and inflammation with fiber (amylose) supplementation. While dietary patterns that predict systemic inflammation have indeed been associated with the risk of CKD [20, 21], as well as the speed of CKD progression [22], it is unknown whether plant-based diets may associate with lower inflammation in these patients.

The purpose of this study was to evaluate whether adherence to plant-based dietary patterns in persons with non-dialysis CKD stages 3–5 is associated with measures of insulin sensitivity and inflammation.

## Materials and methods

### Study population

This investigation was performed in the Uppsala Longitudinal Study of Adult Men (ULSAM) (http://www2.pubcare.uu.se/ULSAM/). The study, initiated in the 1970s, invited all 50-year-old men living in the Uppsala region to participate, and subsequent re-examinations were planned. The present analyses are based on the third examination cycle of the ULSAM cohort. During this examination, participants were 70–71 years of age (examinations performed during 1991–1995; n = 1221), and a detailed comorbid history, risk factor assessment and dietary records were collected simultaneously. For this specific analysis, we set some a priori exclusions: missing cystatin C to calculate GFR or having eGFR >60 mL/min/1.73 m [2] as previously described [23] (n = 594); missing 7-day dietary records or reporting extreme energy intake <800 or >3800 kcal (n = 121); having diabetes at the time of examination (defined as fasting plasma glucose ≥7.0 mmol/L, 2 h post-load glucose level ≥11.1 mmol/L, or the use of oral hypoglycaemic agents or insulin; n = 88). The present study therefore comprises 418 diabetes-free participants with CKD 3–5 and adequate dietary data reporting. None of the participants had a diagnosis of CKD or reported to have CKD, and thus we assume that they were not receiving any nephrology-specific dietetic advice. All participants gave written informed consent, and the Ethics Committee of Uppsala University approved the study protocols.

### Study exposure: adherence to a plant-based diet index

All participants responded 7-day dietary records, using an optical readable form (OMR). The food record used was a pre-coded menu-book, prepared and previously used by the National Food Administration (NFA) [24]. Participants were given oral instructions by a dietitian on how to perform the dietary registration, and the amounts consumed were reported in household measurements or specified as portion sizes according. Nutrient consumption was standardized for total energy intake by regression analysis of the residual method [25]. Macronutrient intake as expressed as percent of energy, and consumption of potassium and fiber as grams per 1000 kcal. We also calculated average daily energy intake (DEI) kcal/kg/day, daily protein intake (DPI) g/kg/day.

Information from food records was used to compute the plant-based diet index (PBDi), as described previously [26]. Briefly, the PBDi is built by scoring the collective intake of plant and animal foods. The intake (in g/day) of 9 plant foods (cereal, fruit, vegetables, coffee and tea, refined grains, potato, juice, jam-sweet drinks and desserts, and chocolate-sweets-sugar) were transformed into quintiles of distribution. The sum of quintile values across these 9 food groups were scored positively (assigning a value of 1 for the first quintile, 2 for the second quintile, 3 for the third quintile, 4 for the fourth quintile and 5 for the fifth quintile). The intake (in g/day) of 5 animal food groups (meat, fish, egg, spreads, and dairy products—ice cream, cheese, milk) was transformed into quintiles of distribution. The sum of quintile values across these 5 food groups were scored reversely (assigning a value of 5 for the first quintile, 4 for the second quintile, 3 for the third quintile, 2 for the fourth quintile and 1 for the fifth quintile). The collective sum of these quintiles reflects the adherence to a plant-based dietary pattern with final scores ranging from 14 (lowest adherence) to 70 (highest adherence).

### Study outcomes

The first study outcome are measures of insulin sensitivity. The gold standard hyperinsulinemic euglycemic glucose clamp was performed as detailed by DeFronzo et al*.* [27] with slight modifications described elsewhere [28]. In brief, two intravenous infusion lines were placed, one into an antecubital vein and the other into a hand or wrist vein. After a 10-min priming infusion, insulin (Actrapid Human; Novo, Copenhagen, Denmark) infusion was held constant at a concentration of 660 pmol/L for 120 min. Plasma glucose was measured every 5 min to be clamped at the euglycemic level (5.1 mmol/L) by infusion of variable amounts of 20% dextrose solution. The total body glucose disposal rate (M value), which is the basic clamp-derived IS index, was the average value of the glucose infusion rate during the final 60 min of the 120-min study (steady state). In addition, the Insulin Sensitivity Index (M/I ratio) was also calculated, which accounts for the insulin concentrations during the last 60 min of the clamp, and thus represents the amount of glucose metabolized (that is, taken up by the body) per unit of plasma insulin and is given in mg/kg^/^min per mU/L of insulin multiplied by 100. The clamp measurement in this cohort is considered to be reliable, as replication tests showed a high intra-individual variation of 0.93 and low coefficient of variation of 0.12.

The second study outcome are measures of systemic inflammation. To this end, blood samples were drawn in the morning after overnight fasting. Plasma and serum determinations were performed at the Department of Clinical Chemistry, University Hospital, Uppsala, which is accredited according to the Swedish Board for Accreditation and Conformity Assessment (Swedac) standard ISO/IEC 17025. C-reactive protein (CRP) measurements were performed by latex enhanced reagent using a Behring BN ProSpec analyzer. The intra-assay coefficient of variation of the CRP method was 1.4% at both 1.23 and 5.49 mg/L. Interleukina-6 (IL-6) was measured by an ELISA kit (IL-6 HS, R&D Systems, Minneapolis, MN. The interassay coefficient of variation was 5%.

### Study covariates

Other study covariates were collected under standardized conditions, including anthropometric and biochemical measurements, and questionnaires regarding medical history, smoking habits, and physical activity level [29]. Body mass index (BMI) was calculated as body weight in kilograms divided by the square of height in meters. The waist and hip circumferences were measured in the supine position. The waist was measured midway between the lowest rib and the iliac crest and the hip over the widest part. The waist/hip ratio was calculated. The use of lipid-lowering and antihypertensive medications were collected with a medical questionnaire according to the, at that time, list of pharmaceutical specialties is available in Sweden (FASS 1992/1993). Insulin concentrations were determined using the Access Immunoassay System (Beckman-Coulter), which uses a chemiluminescent immunoenzymatic assay and Plasma glucose in samples from the oral glucose tolerance test was measured by the glucose dehydrogenase method (Gluc-DH, Merck, Darmstadt, Germany). Blood pressure measuring was attached to the subjects’ non-dominant arm by a skilled lab technician. Systolic (SBP) and diastolic (DBP) blood pressures were measured every 30 min during daytime (06:00–23:00) and every hour during nighttime over 24 h. Hyperlipidaemia was defined as serum cholesterol >5.2 mmol/L, triglycerides >1.71 mmol/L or treatment with lipid-lowering medications. Smoking status was self-reported and defined as current smoking versus non-smoking. Physical activity was self-reported and classified as sedentary and moderate vs regular and athletic [30]. Hypertension was defined as 24-h SBP ≥130 mmHg, 24-h DBP ≥80 mm Hg, or use of antihypertensive medications [31]. CVD comorbid history was obtained by linkage to the Swedish Patient Register (ICD-9 codes 390–459, ICD-10 codes I00-I99).

### Statistical analysis

Values are expressed as mean and standard deviation (SD) for continuous variables with normal distribution, median (interquartile range, IQR) for non-normal distribution variables and percentage of total for categorical. We report baseline characteristics of the sample according to quintiles (Q) of plant based diet score and evaluate P for linear trends across these groups.

We used linear regression models to evaluate the association between PBDi (as a continuous variable, per score unit increase) and study outcomes. Because of skewed data distribution, eGFR, CRP, M and M/I were log-transformed before entering in the regression. Selection of covariates was done on the basis of biological consideration as confounders in the association of interest. Three stepwise models were investigated; In model 1, we considered multivariable adjustment for age and eGFR; In model 2, we further adjusted for total energy intake, lifestyle factors (physical activity, smoking and alcohol intake), BMI and comorbidities (CVD history and hypertension); in model 3, we further adjusted for the use of antihypertensive medication and lipid lowering agents.Finally, we investigated the relationship between PBDi and study outcomes graphically by the use of restricted cubic spline graphs with four degrees of freedom. Data are expressed as regression coefficients and 95% confidence interval (95% CIs). All statistical analysis were performed using STATA software (version 15.1; Stata Corp, College Station, TX).

## Results

### Baseline characteristics

After applying inclusion and exclusion criteria, the cohort consisted of 418 men free from diabetes, aged 70–71 years and with CKD stages 3–5. The majority had CKD stage 3a (n = 322), followed by 3b (n = 86) and 4–5 (n = 10). The most common comorbidities were hypertension (77%) and CVD (36%).

Information from 7-day food records was used to compute the adherence to a PBDi. The PBDi ranged from 14 to 55, with a median of 38. Across increasing quintiles of PBDi (i.e. higher adherence, Table 1), participants showed a lower proportion of smokers, lower alcohol intake, lower BMI, waist circumference, and waist hip ratio, lower proportion of patients with hypertension, lower systolic blood pressure (P < 0.05 for all) and a trend towards higher eGFR (P = 0.06). No significant differences were found for physical activity, CVD history, glucose or insulinIn crude analysis, participants exhibited lower inflammation values (both CRP and IL-6) as well as higher both glucose disposal rate and insulin sensitivity (P < 0.05 for both, Table 1). From a nutritional point of view, as expected, the intakes of fruit, vegetables, carbohydrates, potassium and fiber were greater with higher PBDi quintiles and the intake of fat and animal foods progressively lower. Notably no differences in protein intake were observed (Table 2).

**Table 1:** Baseline characteristics according to quintiles of plant-based diet index (PBDi) distribution

Characteristic	Quintile 1 (n = 78)	Quintile 2 (n = 83)	Quintile 3 (n = 82)	Quintile 4 (n = 91)	Quintile 5 (n = 84)	P trend
PBDi score	31 (14–33)	35 (34–36)	38 (37–39)	41 (40–43)	46 (44–55)	
Age, years	71 ± 0.63	71 ± 0.63	71 ± 0.56	71 ± 0.52	71 ± 0.50	0.19
BMI, kg/m^2^	26.7 ± 3.8	26.2 ± 3.8	26.0 ± 2.7	26.2 ± 3.0	25.4 ± 3.0	0.05
Waist circumference, cm	95.5 (90–104)	95 (88–101)	94 (88–99)	94 (89–99)	93 (87–98)	0.02
Hip circumference, cm	101 (97–106)	100 (96–104)	99 (96–103)	99 (97–103)	100 (95–104)	0.22
Waist/hip ratio	0.96 (0.91–0.99)	0.96 (0.9–0.98)	0.93 (0.9–0.96)	0.95 (0.91–0.98)	0.93 (0.9–0.96)	0.03
Current smokers, n (%)	29 (38)	22 (27)	17 (21)	20 (22)	13 (16)	< 0.01
Sedentary/moderate physical activity, n (%)	38 (49)	37 (45)	30 (37)	35 (39)	34 (41)	0.19
Alcohol intake, g/day	6.3 (1.9–11.1)	4.6 (1.4–11.9)	4.4 (1.0–8.2)	4.4 (1.0–8.0)	2.7 (0.6–6.9)	< 0.01
Cardiovascular disease, %	30 (39)	37 (45)	28 (34)	31 (34)	24 (29)	0.07
Hypertension, n (%)	65 (83)	66 (80)	63 (77)	69 (76)	58 (69)	0.03
ACEI/ARBs, n (%)	6 (8)	11 (13)	8 (10)	6 (7)	5 (6)	0.23
Beta blockers, n (%)	22 (30)	17 (21)	14 (17)	21 (23)	19 (23)	0.47
Calcium channel antagonist, n (%)	13 (18)	10 (12)	5 (6)	14 (15)	10 (12)	0.55
Diuretics, n (%)	13 (18)	14 (17)	9 (11)	14 (15)	8 (10)	0.15
Lipid-lowering drugs, n (%)	6 (8)	8 (10)	7 (9)	14 (15)	7 (8)	0.56
Biochemical measurements						
eGFR, mL/min per 1.73m^2^	50 (44–55)	51 (45–57)	51 (46–56)	51 (47–57)	54 (49–57)	0.06
Sistolic blood pressure	140 ± 17	135 ± 15	135 ± 15	135 ± 16	132 ± 15	0.01
Diastolic blood pressure	77 ± 8	76 ± 9	76 ± 7	76 ± 8	75 ± 8	0.07
Hyperlipidemia, n (%)	59 (76)	63 (76)	63 (77)	69 (76)	65 (77)	0.82
CRP, mg/L	2.6 (1.5–5.5)	2.4 (1.5–4.7)	2.2 (1.2–4.6)	2.1 (1.1–4.4)	1.6 (0.7–4.1)	< 0.01
IL-6 ng/L	4.5 (2.5–9.2)	4.1 (2.4–7.4)	3.7 (2.2–5.9)	3.4 (2.1–5.2)	3.7 (2.1–7.4)	0.03
Glucose, mmol/L	97.3 ± 8.8	95.0 ± 9.9	95.8 ± 8.2	95.0 ± 9.5	96.4 ± 10.1	0.53
Insulin, µg/L	43 (29–62)	45 (29–67)	43 (29–61)	41 (28–60)	42 (32–54)	0.21
Glucose disposal rate	4.9 (3.6–5.9)	5.3 (4.1–6.8)	5.5 (4.4–6.6)	5.2 (3.9–7.0)	5.6 (4.2–6.7)	0.03
Insulin sensitivity, mg/min/kg M/I, 100 × mg/kg/min mU/L	4.4 (3.1–5.9)	4.6 (3.4–6.5)	5.3 (3.9–6.3)	4.6 (3.4–6.9)	5.5 (3.7–7.1)	0.03

Data are expressed as mean ± SD, median (25th, 75th centile), or number (%), as appropriate

*BMI* body mass index, *ACE inhibitors* angiotensin-converting-enzyme inhibitors, *CRP* C-reactive protein, *Insulin sensitivity index (M/I)* 100 × mg/kg/min mU/L

**Table 2:** Nutrient intake characteristics according to quintiles of plant-based diet index (PBDi) distribution

Characteristic	Quintile 1 (n = 78)	Quintile 2 (n = 83)	Quintile 3 (n = 82)	Quintile 4 (n = 91)	Quintile 5 (n = 84)	P trend
PBDi score	31 (14–33)	35 (34–36)	38 (37–39)	41 (40–43)	46 (44–55)	
Total energy intake, kcal/day	1690 (1437–1860)	1691 (1333–1959)	1621 (1294–1876)	1688 (1395–1962)	1806 (1503–2056)	0.06
Dietary energy intake, kcal/kg/day	21 (17–25)	21 (16–25)	20 (16–24)	21 (17–25)	23.5 (19–28)	0.02
Dietary protein intake, g/kg/day	0.8 (0.7–0.1)	0.8 (0.6–1)	0.8 (0.6–0.9)	0.7 (0.6–0.9)	0.8 (0.7–0.97)	0.32
Total carbohydrates, % energy	44.4 (40.3–47.2)	45.2 (42.7–48.9)	48.1 (45.2–51.4)	48.8 (45.2–51.4)	52.2 (49.7–55.7)	<0.01
Total protein, % energy	15.8 (14.3–17.2)	15.6 (13.9–17.2)	15.5 (14.1–16.7)	15.4 (13.9–16.7)	14.9 (13.6–15.8)	< 0.01
Fat, % energy	36.7 (33.1–41.9)	36.2 (31.7–38.4)	34.3 (30.8–37.5)	33.8 (30.3–35.8)	30.9 (28.4–33.3)	< 0.01
Saturated, %	16 (14–18)	15 (14–17)	15 (13–16)	15 (13–16)	13 (12–14)	< 0.01
Monoinsaturared, %	12 (11–15)	13 (11–14)	12 (10–13)	12 (11.13)	11 (10.5–13)	< 0.01
Polyunsaturated, %	5 (4–6)	5 (4–6)	5 (4–6)	5 (4–5)	5 (4–5)	< 0.01
Potassium, mg/1000 kcal	1496 (1320–1692)	1532 (1350–1744)	1559 (1419–1722)	1611 (1476–1818)	1640 (1452–1839)	< 0.01
Fiber intake, g/1000 kcal	8.3 (6.9–9.5)	8.5 (7.8–10.2)	9.5 (8.4–10.8)	9.8 (8.3–11.4)	10.6 (9.3–12.1)	< 0.01
Foog groups g/1000 kcal						
Meat	63 (47–79)	57 (43–79)	49 (39–65)	49 (40–68)	46 (36–56)	< 0.01
Dairy products	248 (168–341)	216 (141–284)	209 (161–285)	195 (148–253)	166 (119–229)	< 0.01
Egg	13 (8–26)	11 (7–17)	7 (4–19)	10 (5–14)	6 (4–11)	< 0.1
Fruit	48 (17–75)	48 (27–76)	71 (30–112)	82 (40–109)	86 (45–128)	< 0.01
Vegetable	29 (14–44)	40 (18–63)	35 (20–53)	46 (28–64)	48 (27–67)	< 0.01

Data are expressed as mean ± SD, median (25th, 75th centile), or number (%), as appropriate

*BMI* body mass index, *ACE inhibitors* angiotensin-converting-enzyme inhibitors, *CRP* C-reactive protein, *Insulin sensitivity index (M/I)* 100 × mg/kg^−1^/min^−1^ mU/L

## *PBDi, insulin sensitivity and inflammation*.

**Fig. 1:**
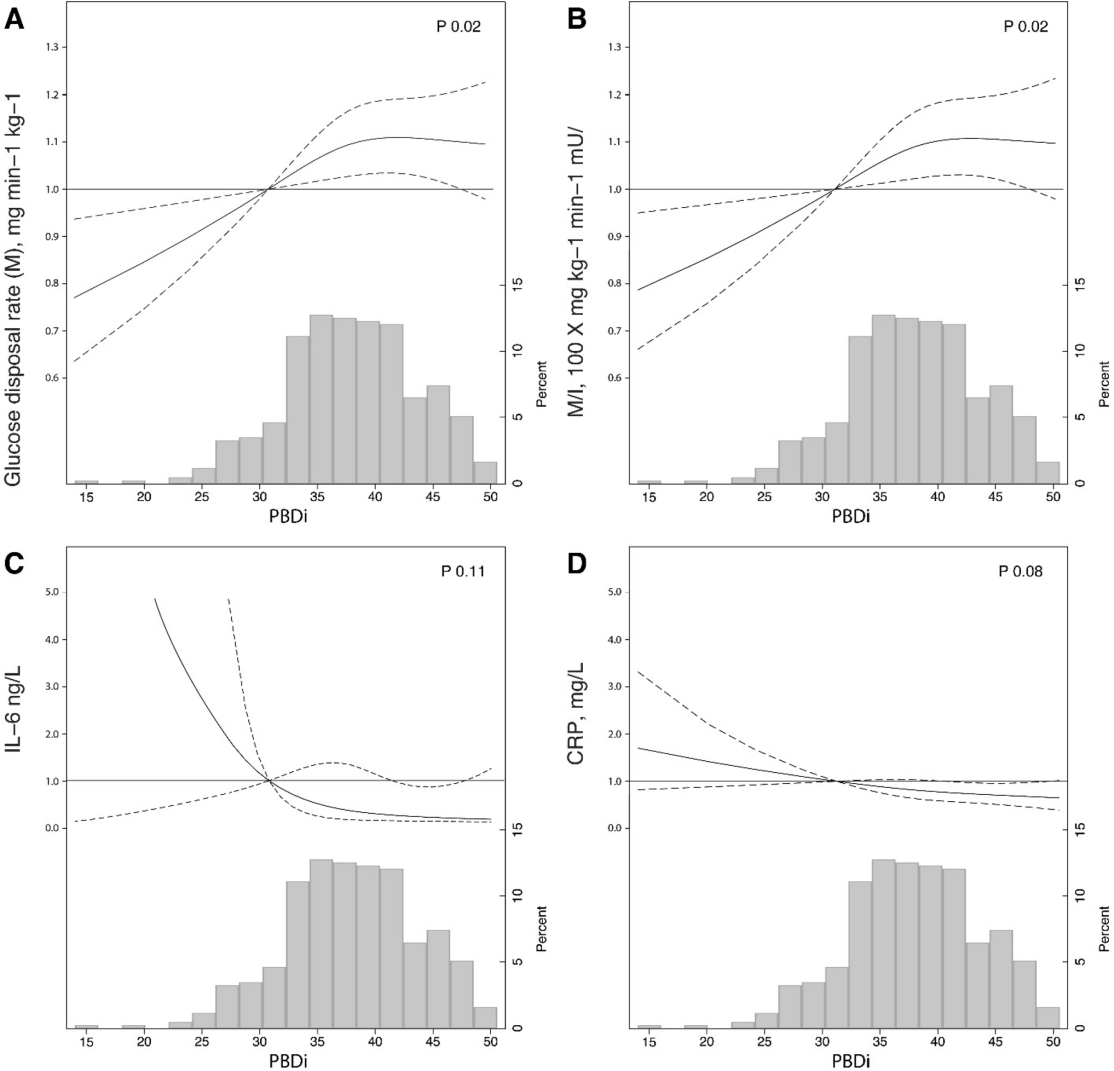
Restricted cubic spline curves showing adjusted β coefficient (bold line) and 95% confidence intervals (dashed lines) for glucose disposal (**a**), insulin sensitivity indes (M/I, **b**), Il-6 (**c**) and CRP (**d**) associated with a plant-based diet index (PBDi) score. Covariates include age, eGFR, total energy intake, physical activity, smoking status, alcohol intake, BMI, CVD history, blood pressure, antihypertensives and lipid lowering medication

We explored whether the association between PBDi score (as a continuous variable) and study outcomes was explained by confounders **(**Table 3**)**. After adjustment for identified confounders in multivariable regression, every unit increase in the PBDi score remained associated with higher glucose disposal rate [β (95% CI) 0.007 (0.001–0.01)] and insulin sensitivity index [β (95% CI) 0.008 (0.001–0.02)]. The fully adjusted model explained 42 and 39% of the variance of these outcomes, respectively. Accordingly, each unit increase in the PBDi was associated with lower levels of IL-6 [β (95% CI) − 0.17 (− 0.33 to − 0.001)] and CRP [β (95% CI) − 0.02 (− 0.04 to − 0.002)]. The shape of these associations are depicted graphically in Fig. 1; although impacted by broad confidence intervals, lower PBDi scores appear associated with worse insulin sensitivity and, more weakly, with increased measures of inflammation.

**Table 3:** Multivariable adjusted linear regression models testing the association between the PBDi score (continuous variable) and measures of insulin sensitivity or systemic inflammation

	Model 1				Model 2				Model 3			
	β coefficient	(95% CI)		P value	β coefficient	(95% CI)		P value	β coefficient	(95% CI)		P value
		Lower	Upper			Lower	Upper			Lower	Upper	
Glucose disposal rate (M), mg min^−1^ kg^−1^	0.008	0.001	0.01	0.02	0.007	0.001	0.01	0.02	0.007	0.001	0.01	0.02
Insulin sensitivity (M/I), 100 × mg kg^−1^ min^−1^ mU/L	0.009	0.002	0.02	0.01	0.008	0.001	0.02	0.03	0.008	0.001	0.02	0.02
Il-6, ng/L	− 0.14	− 0.28	− 0.002	<0.05	− 0.16	− 0.32	− 0.01	0.04	− 0.17	− 0.33	− 0.001	0.04
CRP, mg/L	− 0.02	− 0.04	− 0.01	<0.01	− 0.02	− 0.03	− 0.001	0.04	− 0.02	− 0.04	− 0.002	0.03

Glucose disposal rate, insulin sensitivity, CRP and eGFR were log transformed before entering in the models. Model 1 was adjusted age and eGFR; Model 2: Model 1 + total energy intake, physical activity smoking, alcohol intake, BMI, CVD history and blood pressure; Model 3: Model 2 + antihypertensive and lipid lowering medication

## Discussion

In this observational study we show that among elderly men with non-dialysis CKD stages 3–5 free from diabetes, adherence to a PBDi was moderatedly associated with higher insulin sensitivity and lower systemic inflammation, suggesting a possible role of these diets in the prevention of metabolic complications in CKD.

A number of observational reports in the general population describe improved insulin sensitivity in persons consuming more plant-based foods [32–34], mostly attributed to the intake of cereals and fiber [35, 36]. A recent systematic review [37] reported consistency in the evidence relating vegetarianism with improved glycemic control and fasting glucose levels. In obese mice, the addition of soluble fiber to a high-fat diet significantly improved insulin sensitivity [38]. Our findings agree with this evidence and expand it to patients with CKD. Early small sample-size but well-designed studies observed improved insulin sensitivity as assessed by the clamp technique among patients with non-dialysis dependent CKD who followed a quasi-vegetarian very low protein diet supplemented with ketoanaloques [39, 40]. More recentry, a 4-week trial [15] of fermentable fiber supplementation in persons advanced CKD failed however to observe changes in insulin resistance. We argue that the small sample size (n = 39), the narrow range of eGFR included (15–25 mL/min), the short-term nature of the study and the surrogacy of the outcome (HOMA-IR) may have been responsible for this lack of association. Adherence to plant-based diets implies, by definition, a progressively lower intake of meat protein, which may have additional benefits for delaying ESKD [41]. In this sense, we note that diets rich in animal foods have been shown to increase endogenous acid production with subsequent metabolic acidosis [42] and insulin resistance [43]. Evidence from a crossover trial of postmenopausal women suggests that replacing meat with soy results in improved insulin sensitivity [44]. In persons with CKD stages: 3–5, soy-based diets have been used as prototypes for low protein diets, documenting improved gut microbial profile [45, 46].

Single nutrients commonly rich in plant-based diets have been associated inversely with measures of systemic inflammation. Observational studies in persons with CKD [16, 47] reported that higher fiber intake was associated with lower both CRP and IL-6 levels. In a small interventional study, addition of fiber supplements (inulin) to a low protein diet resulted in reduced CRP levels in patients with CKD compared to a low protein diet alone [48]. Similar results were observed in animals [18] and in persons with CKD [19] supplemented with amylose. Our results of an association between the overall PBDi score and inflammation are novel, but agree with and expand this single-nutrient evidence. Mediterranean diets are also examples of a plant-based diet, as they promote a moderate consumption of animal food and a higher proportion of plant-foods. Landmark trials in the general population showed that Mediterranean diets can reduce markers of inflammation [49] and lower the risk of major cardiovascular events [50]. Evidence in CKD is scarce; in 40 persons CKD G2, a 90-day intervention of a Mediterranean diet [51] resulted in improvements in blood lipids and reductions in levels of CRP.

Strengths of our study include the stringent population selection (men of similar age), ascertainments of exposure from 7-day food records and of outcomes by gold-standard methods, particularly insulin sensitivity. Although we used a established plant-based diet scoring, there are other scores and dietary patterns in the literature [52] and this limits comparison with other studies. We acknowledge that our food composition software did not allow the separation of legumes and nuts, which may have reduced the sensitivity of our scoring, nor give distinct consideration of less-healthy plant foods such as potatoes (especially consumed as French fries) and added sugars. Associations are cross-sectional and sex- and cohort-specific in a population of men of Nordic upbringing and free from diabetes. Extrapolation to women, other diseases, regions and periods should be done with caution. A priori, we do not anticipate associations to differ between sexes. However, aging encompasses changes in inflammation markers, physical activity and/or frailty. Studies evaluating these associations in women as well as in younger populations should follow. Persons with a higher PBDi score also had other typical features of healthy lifestyle, such as less smoking, being more physically active, consuming less alcohol or having a lower BMI. A higher PBDi score may also reflect socioeconomic differences. Although we tried to adjust for some of these factors in our multivariable analyses, a limitation of this and any observational study is the presence of unknown confounding our inability to establish causation. The results may to some extent reflect a more conscious attitude to lifestyle risks in general among those with a lower PBDi score. We recognize that the risk magnitude of our associations, although statistically significant, is not big and (as evidenced by the spline curves) is affected by broad confidence intervals. This can be attributed to a relatively low sample size in our cohort, but also perhaps to the fact that diet is one of many and probably not the most important factor leading to insulin resistance and inflammation in such polymorbid and complex patients.

To date, only one controlled study in patients with macroalbuminuria and CKD G3 has explored the effect of increasing the intake of fruits and vegetables. Randomization for three years to usual care or interventions designed to reduce dietary acid by 50%, using either sodium bicarbonate or alkali-rich fruits and vegetables [9] resulted in similar control of metabolic acidosis and eGFR preservation and to a greater extent than usual care. Of note, a 5-year extension of the follow up of this trial [53] not only confirmed these findings, but also showed that the group receiving fruits and vegetables had better systolic blood pressure and body mass index control, improved lipid profile and higher serum levels of vitamin K_1_ than those under bicarbonate therapy or usual care [53].

To conclude, adherence to a plant-based diet, as represented through the PBDi score, was associated with higher insulin sensitivity and lower systemic inflammation in elderly men with CKD stages 3–5. Such findings suggest a possible role of these diets in the prevention of metabolic complications for these patients, and adds to recent observational studies associating plant-based diets with a lower risk of death and progression to ESKD. Whether the associations are causal can only be answered in an adequately designed and powered interventional study.
